# A Nanosized Porous Supramolecular Lead(II)–*N*′-phenyl(pyridin-2-yl)methylene-*N*-phenylthiosemicarbazide Aggregate, Obtained Under Electrochemical Conditions

**DOI:** 10.1021/acs.inorgchem.4c02182

**Published:** 2024-09-26

**Authors:** Ghodrat Mahmoudi, Isabel Garcia-Santos, Elena Labisbal, Alfonso Castiñeiras, Vali Alizadeh, Rosa M. Gomila, Antonio Frontera, Damir A. Safin

**Affiliations:** † Department of Chemistry, Faculty of Science, University of Maragheh, Maragheh 55136-83111, Iran; ‡ Chemistry Department, Faculty of Engineering and Natural Sciences, Istinye University, Sarıyer, Istanbul 34396, Turkey; § Department of Technical Sciences, Western Caspian University, Baku 1001, Azerbaijan; ∥ Departamento de Química Inorgánica, Facultad de Farmacia, Universidad de Santiago de Compostela, Santiago de Compostela E-15782, Spain; ⊥ Department of Petroleum Engineering, Faculty of Engineering, University of Garmsar, Garmsar 3581755796, Iran; # Departament de Química, Universitat de les Illes Balears, Crta de Valldemossa km 7.5, Palma de Mallorca 07122, Spain; ∇ University of Tyumen, Volodarskogo Str. 6, Tyumen 625003, Russian Federation; ○ Scientific and Educational and Innovation Center for Chemical and Pharmaceutical Technologies, Ural Federal University named after the First President of Russia B.N. Yeltsin, Mira Str. 19, Ekaterinburg 620002, Russian Federation

## Abstract

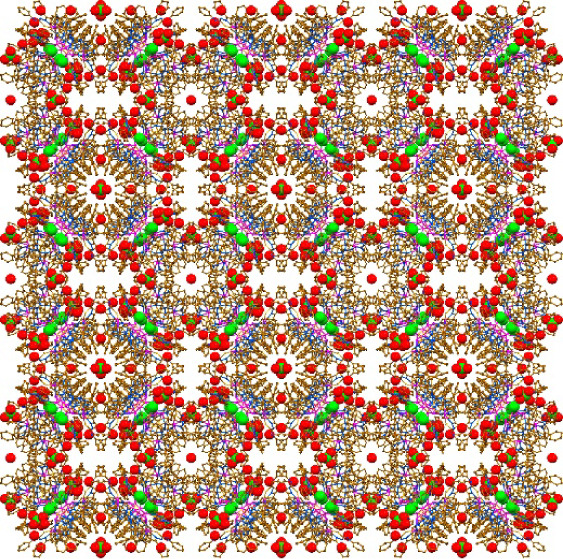

A novel nanosized porous supramolecular nonanuclear complex [Pb_9_(HL)_12_Cl_2_(ClO_4_)](ClO_4_)_3_·15H_2_O*·a*(solvent) (**1**·15H_2_O*·a*(solvent)) is reported that was synthesized by electrochemical oxidation of a Pb anode under the ambient conditions in a CH_3_CN:MeOH solution of *N*′-phenyl(pyridin-2-yl)methylene-*N*-phenylthiosemicarbazide (**H**_**2**_**L**), containing [N(CH_3_)_4_]ClO_4_ as a current carrier. The supramolecular aggregate of **1** is enforced by a myriad of Pb···S tetrel bonds (TtBs) established with the thiocarbonyl sulfur atoms of adjacent species, which have been also analyzed by DFT calculations via 2D maps of ELF, Laplacian and RDG properties. Moreover, Pb···Cl TtBs with the central Cl^–^ anion, and Pb···O TtBs with the three oxygen atoms of the ClO_4_^–^ anion, were revealed. Notably, the molecular structure of **1** differs significantly from that recently reported by us [Pb_2_(HL)_2_(CH_3_CN)(ClO_4_)_2_]·2H_2_O (**2**·2H_2_O), which was obtained using a conventional synthetic procedure by reacting Pb(ClO_4_)_2_ with **H**_**2**_**L** in the same CH_3_CN:MeOH solution, thus highlighting a crucial role of the electrochemical conditions. The optical characteristics of the complex were investigated using UV–vis spectroscopy and spectrofluorimetry in methanol. The complex was found to be emissive when excited at 304 nm, producing a broad emission band ranging from approximately 420 to 600 nm with multiple peaks. The CIE-1931 chromaticity coordinates, calculated as (0.33, 0.24), suggest that the emission lies in the white region of the chromaticity diagram. Further investigation is needed to fully characterize the origin of this emission.

## Abstract 2

We report a nanosized porous supramolecular nonanuclear complex [Pb_9_(HL)_12_Cl_2_(ClO_4_)](ClO_4_)_3_•15H_2_O•*a*(solvent) (**1**•15H_2_O•*a*(solvent)), which was obtained by electrochemical oxidation of a lead anode under the ambient conditions in a CH_3_CN:MeOH solution of *N*′-phenyl(pyridin-2-yl)methylene-*N*-phenylthiosemicarbazide (**H_2_L**), containing [N(CH_3_)_4_]ClO_4_ as a current carrier.

## 1 Introduction

A variety of synthetic procedures have actively been used to produce coordination compounds. Of these synthetic approaches, using metal salts as a source of the corresponding metal cations is, likely, the most conventional one. However, the so-called direct synthesis from zerovalent metals has actively been used for the fabrication of coordination compounds.^1^ Of this type of synthesis, electrosynthesis (a synthetic approach under electrochemical conditions), usually, proceeds upon dissolution of the zerovalent metal as an anode of the electrochemical scheme.^2−5^ The advantages of electrochemical reactions in the synthesis of coordination compounds are a one-step reaction, which does not require oxidants or reductants. Furthermore, such a type of reaction allows to use organic solvents, which application is impossible in conventional synthetic methods due to insolubility of starting metal sources (salts). All this allows to obtain coordination compounds with intriguing structures otherwise difficult to produce.

Modern synthetic chemistry utilizes a wide range of noncovalent interactions (NCIs) as essential tools for influencing crystal packing. Among these interactions, hydrogen bonds^6−8^ and π-stacking^9−11^ interactions are particularly significant. Approximately 15 years ago, the concept of the σ-hole was introduced.^12^ Over time, both σ- and π-hole interactions have been recognized as key structure-determining forces in molecular assemblies. In this type of contacts, σ- and π-holes are positive regions on a Lewis acidic atom, which can engage in interactions with electron-rich atoms, acting as Lewis bases (LBs), most commonly a lone pair (LP) donor atom. Among the various types of σ-hole interactions, TtB has gained considerable attention. This specific noncovalent interaction involves a group 14 element functioning as a Lewis acid (LA).^13^ The lead(II) cation (Pb^2+^) is of particular interest in the context of TtB, owing to its variable coordination numbers and large ionic radius. Additionally, the 6s^2^ LP in the Pb^2+^ cation can promote either hemi- or holodirectional coordination,^14−17^ with the former being conducive to tetrel bonding, thus facilitating the formation of supramolecular assemblies with distinctive properties.

With these considerations in mind, and building upon our extensive research into the coordination chemistry of Pb^2+^ architectures and the influence of NCIs in forming extended structures,^18−40^ we focused our attention to *N*′-phenyl(pyridin-2-yl)methylene-*N*-phenylthiosemicarbazide (**H_2_L**),^33^ a ligand intentionally designed as a potentially tridentate chelator with an extended π-system. This ligand was employed in an electrochemical reaction with zerovalent lead as the complexing agent. Interestingly, a thorough search of the Cambridge Structural Database (CSD)^41^ identified just 21 crystal structures involving complexes derived from **H_2_L**, specifically [Pb(HL)NO_3_],^23^ [Pb_2_(HL)_2_(ClO_4_)_2_(CH_3_CN)]·2H_2_O (**2**·2H_2_O),^33^ [Pb(HL)(OAc)],^34^ [SnBu_2_(HL)Cl],^42^ [SnBu(HL)Cl_2_]·H_2_O,^43^ [Sn(HL)Cl_3_]·EtOH,^43^ [SnPh(HL)Cl_2_],^44^ [SnPh_2_(HL)Cl],^44^ [SnMe_2_(HL)(OAc)]·EtOH,^45^ [SnPh_2_(HL)(OAc)]·EtOH,^45^ [Ni(HL)(NCS)],^46^ [Ni(HL)(NCS)]·DMF,^46^ [Ni(HL)N_3_],^46^ [Cu(HL)I],^47^ [Cu(HL)(NCS)],^48^ [Cu(HL)Cl]·H_2_O,^47,48^ [Sb(HL)Cl_2_],^49^ [Pd(HL)Cl],^50,51^ [Zn(HL)_2_]·DMF,^52^ [Au(H_3_L)Cl]Cl^53^ and [Ga(HL)_2_]NO_3_.^54^ Thus, the coordination chemistry of **H**_**2**_**L** was limitedly studied and exclusively in the conventional synthetic procedures.

## 2 Experimental Section

### 2.1 Materials and Physical Measurements

All solvents and reagents utilized were obtained from commercial suppliers and were employed without further purification. **H**_**2**_**L** was synthesized following the procedure recently described in the literature.^33^ FTIR spectra were recorded using KBr pellets on an FT 801 spectrometer. The ^1^H NMR spectrum in DMSO-*d*_6_ was measured using a Bruker DPX FT/NMR-400 spectrometer. The UV–vis and fluorescence spectra were acquired from a recently prepared solution of the sample in recently distilled methanol, utilizing a Jasco V-770 spectrophotometer and an Edinburgh Instruments FS5 spectrofluorometer.

### 2.2 Synthesis

The complex was synthesized via an electrochemical process under ambient conditions. The setup included a tall-form beaker (100 mL) fitted with a rubber bung to allow the electrodes to enter. A solution of **H**_**2**_**L** (0.060 g, 0.18 mmol) in a 1:1 mixture of CH_3_CN:CH_3_OH (80 mL) containing [N(CH_3_)_4_]ClO_4_ as the supporting electrolyte was electrolyzed using a platinum wire as the cathode and a lead metal plate as the sacrificial anode at 6 V and 5 mA for 1 h, resulting in the dissolution of 18 mg of lead (*E*_f_ = 0.50 mol F^–1^). **Caution!***While no issues arose during this work, it should be noted that all ClO*_*4*_^*–*^*compounds are potentially explosive and must be handled with great care and in small quantities!* Orange prism-like crystals, suitable for X-ray diffraction studies, were achieved by evaporation of the solution. H_2_ gas evolved at the cathode during the electrolysis. Under these conditions, the cell can be summarized as Pb_(+)_/**H**_**2**_**L +** CH_3_CN/Pt_(−)_.

### 2.3 X-ray Diffraction Analysis

Single-crystal X-ray diffraction data were obtained at 100(2) K using a Bruker D8 VENTURE PHOTON III-14 diffractometer equipped with Mo–Kα radiation (λ = 0.71073 Å) and a graphite monochromator. The data set was processed through APEX4 software^55^ and absorption corrections were applied using SADABS.^56^ The structure was determined by direct methods via the SHELXS-2013 program^57^ and refined using the full-matrix least-squares technique implemented in SHELXL-2013.^57^ Hydrogen atoms were located in the difference map and treated as fixed contributors attached to their respective atoms, with isotropic thermal parameters set at 1.2 times the value of the carrier atoms. Crystallographic details are as follows: C*_50_*H*_40_*N*_20_*O*_10_*Pb*_4_*, 4(H*_2_*O); *M*_r_ = 24616.47 g/mol; cubic space group *P*–43*n*; *a* = 38.3201(12) Å; *V* = 56270(5) Å^3^; *Z* = 2; ρ = 1.453 g/cm^3^; μ(Mo–Kα) = 5.545 mm^–1^. A total of 213 600 reflections were collected, of which 13 516 were unique (*R*_int_ = 0.083). The final refinement gave *R*_1_(all) = 0.0616, *wR*_2_(all) = 0.0991, and *S* = 1.043. The crystallographic data has been deposited at the Cambridge Crystallographic Data Centre (CCDC 2348952) and can be accessed free of charge via https://www.ccdc.cam.ac.uk/structures or by contacting the CCDC directly.

### 2.4 Theoretical Methods

The trimeric assemblies included herein were modeled using the PBE0-D3/def2-TZVP level of theory^58−60^ with the Gaussian-16 software package,^61^ based on crystallographic coordinates. This approach was selected to focus on the interactions as they exist in the solid state, rather than optimizing for the most stable conformation. This level of theory has been widely and successfully applied in the literature^18,27,29,32,35,40^ to study Pb(II) coordination compounds. An analysis of the electron density using the quantum theory of “atoms-in-molecules” (AIM)^62^ was performed at the same theoretical level, utilizing the Multiwfn software.^63^ Additionally, reduced density gradient (RDG)^64^ and electron localization function (ELF)^65^ 2D plots were generated using the same program.^63^ The Laplacian of the electron density was broken down into contributions along the three principal axes of maximal variation, producing the eigenvalues of the Hessian matrix (λ_1_, λ_2_, and λ_3_). The sign of λ_2_ was employed to differentiate between attractive (λ_2_ < 0) interactions, indicative of bonding, and repulsive (λ_2_ > 0) nonbonding interactions. The sign of λ_2_ is used to distinguish bonding (attractive, λ_2_ < 0) weak interactions from nonbonding (repulsive, λ_2_ > 0) ones.^64^ Periodic boundary conditions (PBC) were not employed in this work, as our analysis focused on QTAIM and NCIplot methods, which are not sensitive to PBC. Additionally, crystallographic coordinates were used for the calculations.

## 3 Results and Discussion

**Scheme 1:**
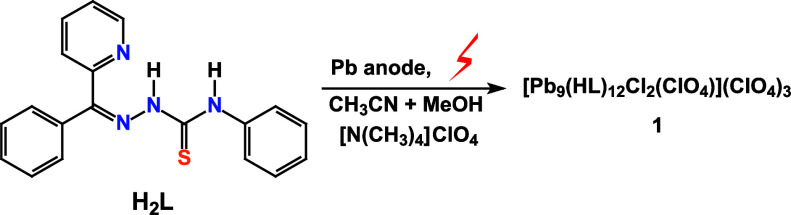
Synthesis **1**

Electrochemical oxidation of a Pb anode under the ambient conditions in a solution of **H**_**2**_**L** in a mixture of acetonitrile and methanol, containing [N(CH_3_)_4_]ClO_4_ as a current carrier, allowed to produce a novel nanosized supramolecular nonanuclear complex [Pb_9_(HL)_12_Cl_2_(ClO_4_)](ClO_4_)_3_·15H_2_O*·a*(solvent) (**1**·15H_2_O*·a*(solvent)) (Scheme 1), which orange prism-like crystals, suitable for X-ray analysis, were obtained by slow evaporation.

**Figure 1:**
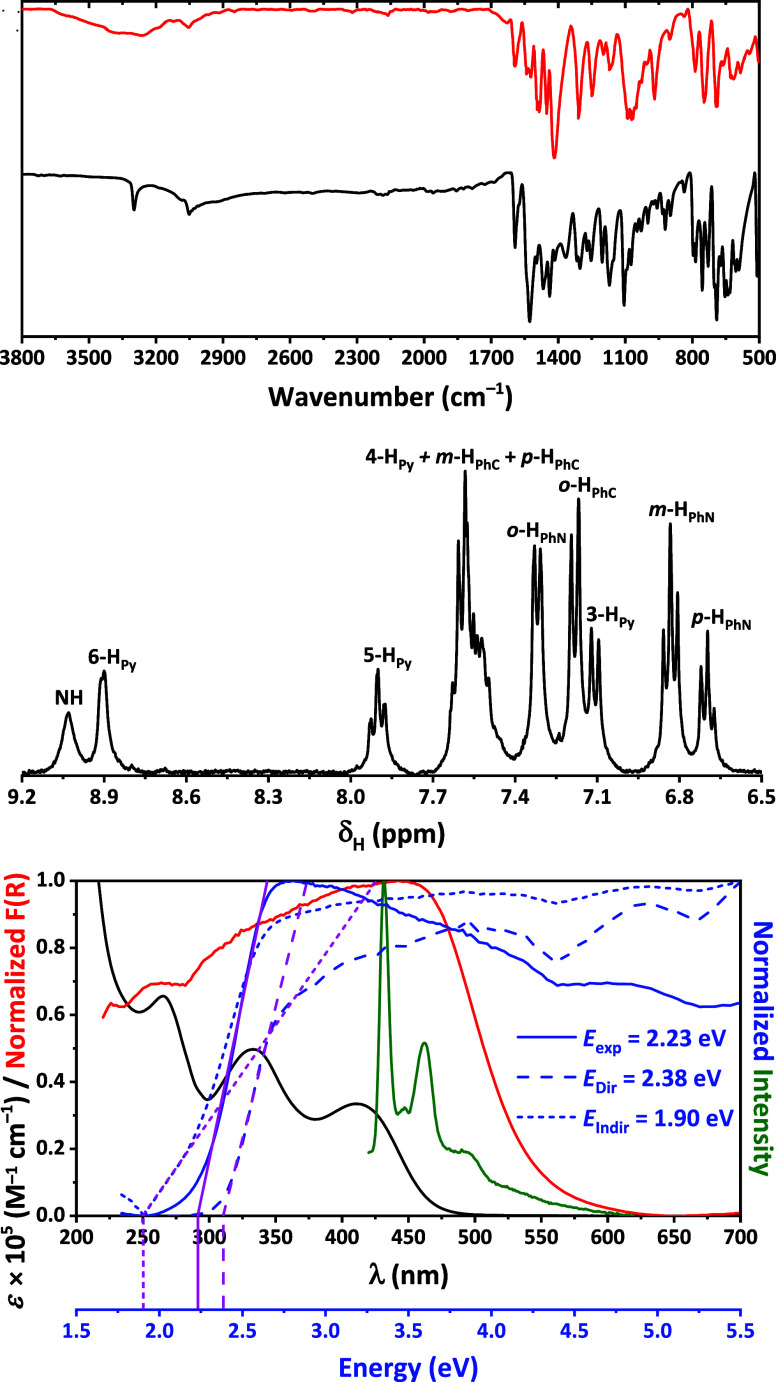
(top) The IR spectra of **H**_**2**_**L** (black) and **1**·15H_2_O·*a*(solvent) (red). (middle) The ^1^H NMR spectrum of **1·**15H_2_O·*a*(solvent) in DMSO-*d*_6_. (bottom) The UV–vis (black) and luminescence (green) in MeOH, normalized Kubelka–Munk (red and solid blue), normalized (*αhν*)^2^ (dashed blue) and (*αhν*)^1/2^ (short dashed blue) spectra of **1**·15H_2_O·*a*(solvent).

A comparison of the FT-IR spectra of **H**_**2**_**L** and the complex indicates the lack of the NH(N) group band, which appears at 3300 cm^–1^ in the **H**_**2**_**L** spectrum (Figure 1), suggesting deprotonation of this group upon Pb^2+^-coordination. The presence of ClO_4_^–^ anions in the complex is confirmed by a broad band centered around 1080 cm^–1^ (Figure 1). Additionally, the complex’s spectrum shows a broad band between 3120–3680 cm^–1^ and a shoulder near 1635 cm^–1^, which are assigned to crystal water molecules. The ^1^H NMR spectrum of the complex in DMSO-*d*_6_ also supports the deprotonated form of the ligand, as it lacks the peak corresponding to the NH(N) hydrogen, which appears at 13.14 ppm in the ^1^H NMR spectrum of **H**_**2**_**L** recorded in the same solvent (Figure 1).^40^ Interestingly, the ^1^H NMR spectrum of the complex displays a single set of peaks (Figure 1), in contrast to **H**_**2**_**L**, which shows two groups of signals corresponding to its *E*- and *Z*-isomers, with the *Z*-isomer being predominant in DMSO-*d*_6_.^40^ The UV–vis spectrum in methanol exhibits bands extending up to 500 nm, with distinct maxima at 265, 333, and 410 nm (Figure 1). The higher energy bands are attributed to intraligand transitions, while the lower energy band is associated with ligand-to-metal charge transfer. The Kubelka–Munk spectrum shows bands extending up to around 625 nm, with estimated direct and indirect band gaps of 2.38 and 1.90 eV, respectively (Figure 1). Notably, the complex is emissive in methanol when excited at 304 nm, producing a broad emission band ranging from approximately 420 to 600 nm, with peaks at ∼430, ∼ 460, and ∼495 nm, and a long tail extending from ∼515 nm (Figure 1). The CIE-1931 chromaticity coordinates of (0.33, 0.24) position the emission in the white region of the chromaticity diagram, suggesting that the complex functions as a single-component white light-emitting phosphor.

Complex **1**·15H_2_O·*a*(solvent) crystallized in cubic space group *P*–43*n* with the crystallographic axis of 38.3201(12) Å. The solvent accessible volume of the structure was calculated to be 15 505 Å^3^, which comprise about 27.6% of the unit cell. It was found that voids are filled with water molecules. Furthermore, additional electron densities were revealed in the voids, which, however, were squeezed.

**Figure 2:**
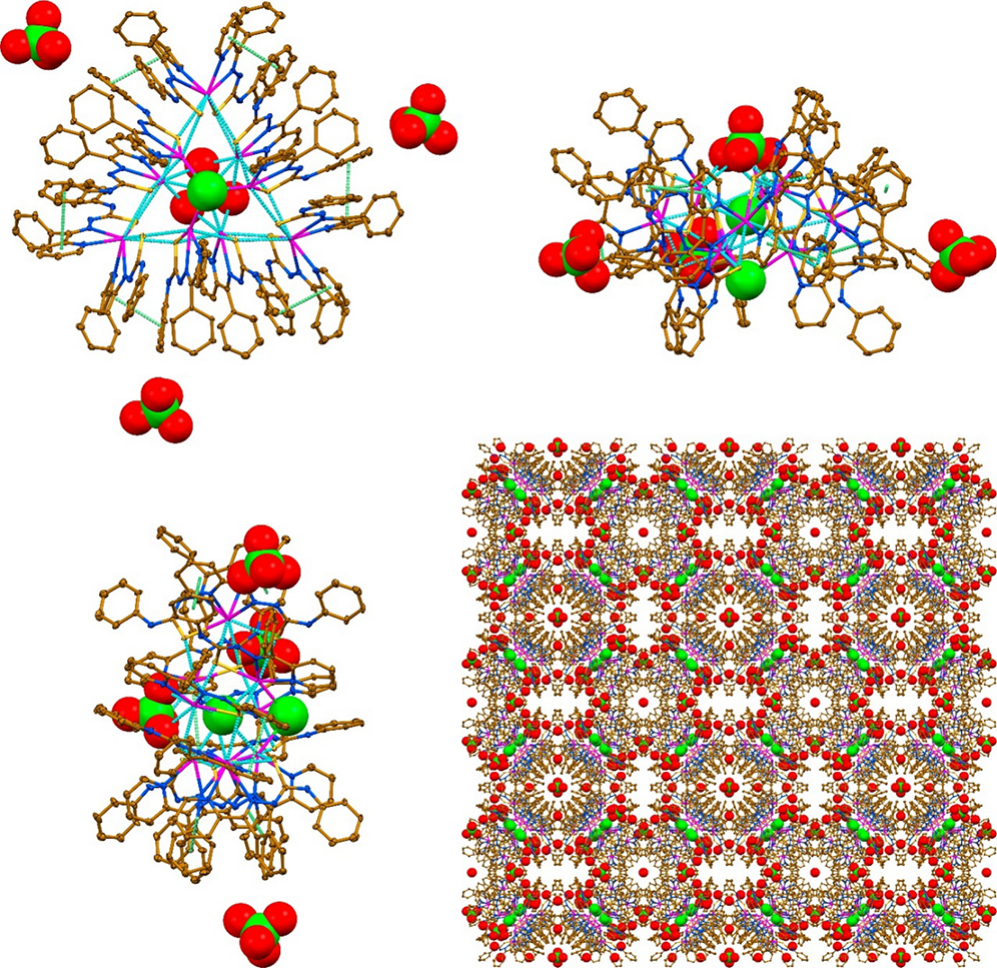
Different views on the molecular structure of **1** and crystal packing of **1**·15H_2_O·*a*(solvent). Hydrogen atoms were omitted for clariy. Color code: C = gold, *N* = blue, O = red, Cl = green, Pb = magenta; Pb···S/O/Cl tetrel bonds = cyan dashed line, π···π interaction = green dashed line.

The supramolecular cationic cluster **1**^3+^ is constructed from three different species, namely a trinuclear cation [Pb_3_(HL)_3_Cl]^2+^ of a triangle shape, three mononuclear neutral species [Pb(HL)_2_] and three mononuclear cations [Pb(HL)]^+^ (Figure 2). In the triangle cationic species each of three space gaps is filled by one [Pb(HL)_2_] and one [Pb(HL)]^+^ species, all together yielding a cyclic supramolecular architecture with a void in the central part (Figure 2). The latter is further filled by an additional Cl^–^ anion, which is located in the middle part of this void, and one ClO_4_^–^ anion, which “seals” this void (Figure 2). The anionic ligands **HL** in each cationic species [Pb_3_(HL)_3_Cl]^2+^ and [Pb(HL)]^+^ as well as one of the ligands in the neutral species [Pb(HL)_2_] covalently link the Pb^2+^ cations through the nitrogen atoms of pyridine and imine, and thiocarbonyl sulfur atom. The second ligand in [Pb(HL)_2_] links the metal cation by two covalent bonds from the pyridine and imine nitrogen atoms, and one tetrel bond from the thiocarbonyl sulfur atom (Table 1).

**Table 1:** Selected Bond Lengths (Å) in Compound **1**^a^

Bond	Length	Type	Bond	Length	Type
Pb1–N31	2.570(15)	covalent	Pb2···S1	3.012(4)	tetrel
Pb1–N32	2.600(15)	covalent	Pb2···S3	3.041(4)	tetrel
Pb1–S3	2.850(4)	covalent	Pb2···Cl5	3.354(4)	tetrel
Pb1···O11	2.990(12)	tetrel	Pb3–N11	2.505(12)	covalent
Pb1···S2	3.027(4)	tetrel	Pb3–N12	2.802(12)	covalent
Pb1···S4	3.032(4)	tetrel	Pb3–N41#^1^	2.600(12)	covalent
Pb1···Cl5	3.231(4)	tetrel	Pb3–N42#^1^	2.693(14)	covalent
Pb2–N21	2.555(15)	covalent	Pb3–S4#^1^	2.902(4)	covalent
Pb2–N22	2.503(12)	covalent	Pb3···S1	3.115(4)	tetrel
Pb2–S2	2.830(5)	covalent	Pb3···S2	3.285(4)	tetrel
Pb2–Cl6	3.153(5)	tetrel	Pb3···S3	3.434(4)	tetrel

a Symmetry code: #1 *z*, *x*, *y*.

The supramolecular aggregate of **1**^3+^ is enforced by a myriad of Pb···S TtBs established with the thiocarbonyl sulfur atoms of adjacent species, Pb···Cl tetrel bonds with the central Cl^–^ anion, and Pb···O TtBs with the three O- atoms of the ClO_4_^–^ anion (Figure 2 and Table 1). The molecular structure of **1**^3+^ is further stabilized by π-stacking interactions between the both pyridine rings of the neutral species [Pb(HL)_2_] and Ph(N) rings of adjacent chelate species (Figure 2 and Table 2). Notably, the molecular structure of **1** differs significantly from that recently reported by us **2**·2H_2_O, which was obtained using a conventional synthetic procedure by reacting Pb(ClO_4_)_2_ with **H**_**2**_**L** in the same CH_3_CN:MeOH solution,^33^ thus highlighting a crucial role of the electrochemical conditions. The reasons for the formation of a different structure under electrochemical conditions stand behind the redox reaction, where the lead anode is oxidized to the Pb^2+^, while the ClO_4_^–^ anion is reduced to the Cl^–^ anion, which, in turn, is involved in the structure of **1**^3+^ playing a crucial template role. Contrarily, under conventional synthetic conditions the metal is directly introduced in the cationic form, and the ClO_4_^–^ anion is not reduced; thus, the Cl^–^ anion is not formed.

**Table 2:** Hydrogen Bond and π-Stacking Lengths (Å) and Angles (°) in the Crystal Structure of **1**·15H_2_O·*a*(Solvent)

D–H···A	*d*(D–H)	*d*(H···A)	*d*(D···A)	∠(DHA)
N44–H44A···O2	0.88	2.08	2.92(2)	159

Cg (*I*)	Cg (*J*)	*d*[Cg(*I*)···Cg(*J*)]	α	β	γ	Slippage
Py_N11_	Ph_C28_	3.913(11)	10.7(9)	14.2	24.4	0.958
Ph_C28_	Py_N11_	3.912(11)	10.7(9)	24.4	14.2	1.614
Py_N41_	Ph_C48_	3.637(9)	14.5(8)	9.2	11.1	0.581
Ph_C48_	Py_N41_	3.636(9)	14.5(8)	11.1	9.2	0.698

The Pb–N_Py_ and Pb–N_imine_ bond lengths in the species [Pb_3_(HL)_3_Cl]^2+^, [Pb(HL)]^+^ and [Pb(HL)_2_] are similar and of 2.503(12)–2.600(15) Å, while the Pb–N_imine_ distances in the latter species are remarkably longer and of 2.693(14) Å and 2.802(12) Å (Table 1). The covalent Pb–S bonds are 2.830(5)–2.902(4) Å, while the tetrel Pb···S bonds vary from 3.012(4) Å to 3.434(4) Å (Table 1). The covalent Pb–Cl bonds in the cationic species [Pb_3_(HL)_3_Cl]^2+^ are 3.153(5) Å, while the tetrel Pb···Cl bonds, formed with the central Cl^–^ anion, are 3.231(4) Å and 3.354(4) Å (Table 1). Finally, the Pb···O TtBs with the O- atoms of the ClO_4_^–^ anion are 2.990(12) Å (Table 1).

**Figure 3:**
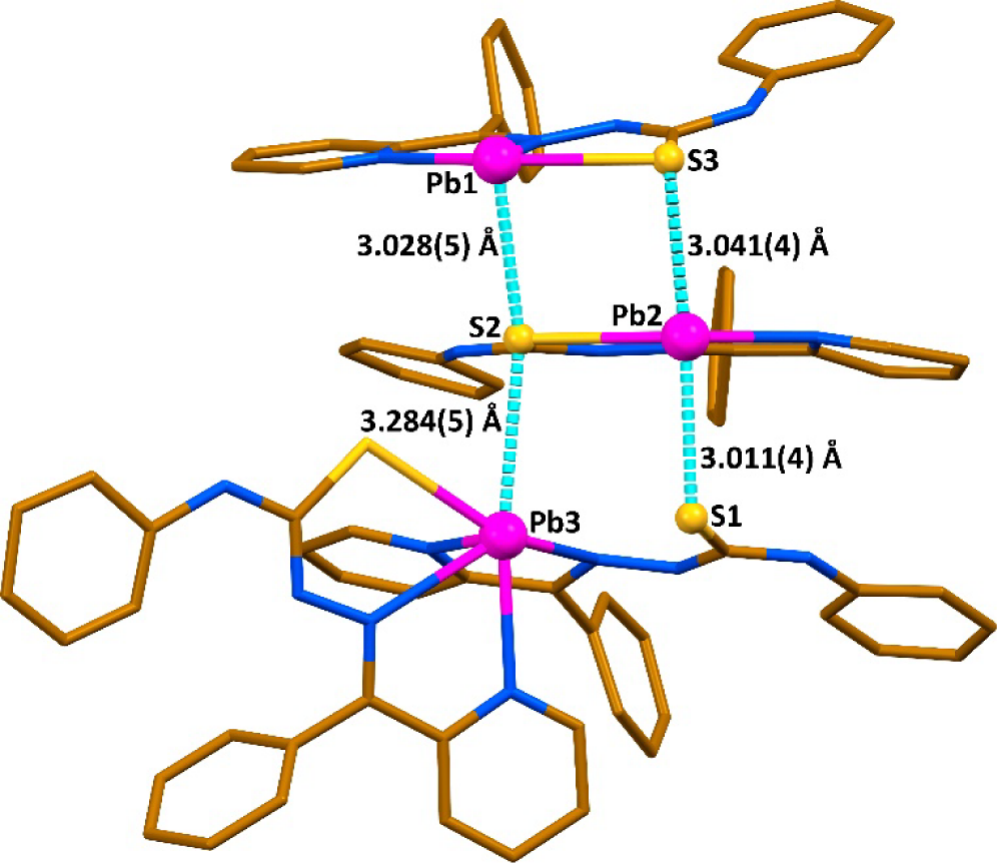
Partial view of the X-ray structure of **1** evidencing the formation of a trimeric assembly by means of Pb···S tetrel bonds.

DFT analysis focused on the study of the intermolecular Pb···S TtBs that are important in determining the crystal packing of **1**. We have analyzed the fragment of the crystal structure, where four different Pb···S TtBs are established with distances that range from 3.012(4) to 3.285(4) Å (Figure 3). For the study we have defined two planes (Pb1/S2/Pb3 and S1/Pb2/S3) to plot the Laplacian of the density (∇^2^ρ), electron localization function (ELF), and reduced density gradient (RDG) 2D plots. These properties combined are useful to analyze the (non)covalent nature of the Pb···S contacts. The ∇^2^ρ 2D plot offers insights into the covalent nature of the NCI, while the RDG maps are effective in pinpointing regions where noncovalent interactions occur. Additionally, the ELF 2D map was employed to distinguish between LB and LA regions within the TtB trimer (Figure 3). This analysis is further supported by the BCP parameters (Table 3), which shed light on the stability of the Pb···S TtBs within the trimer.

**Table 3:** QTAIM and ELF Values (a.u.) for the BCPs Connecting the Pb and S Atoms that Characterize the Tetrel Bonds in the Structure of **1**

BCP	ρ (r)	G (r)	V (r)	∇^2^ρ (r)	ELF	λ_2_
Pb1···S2	0.0284	0.0146	–0.0168	0.0496	0.21	–0.022
Pb3···S2	0.0181	0.0081	–0.0082	0.0318	0.16	–0.013
Pb2···S1	0.0310	0.0156	–0.0187	0.0503	0.24	–0.024
Pb2···S3	0.0289	0.0148	–0.0170	0.0494	0.22	–0.022

**Figure 4:**
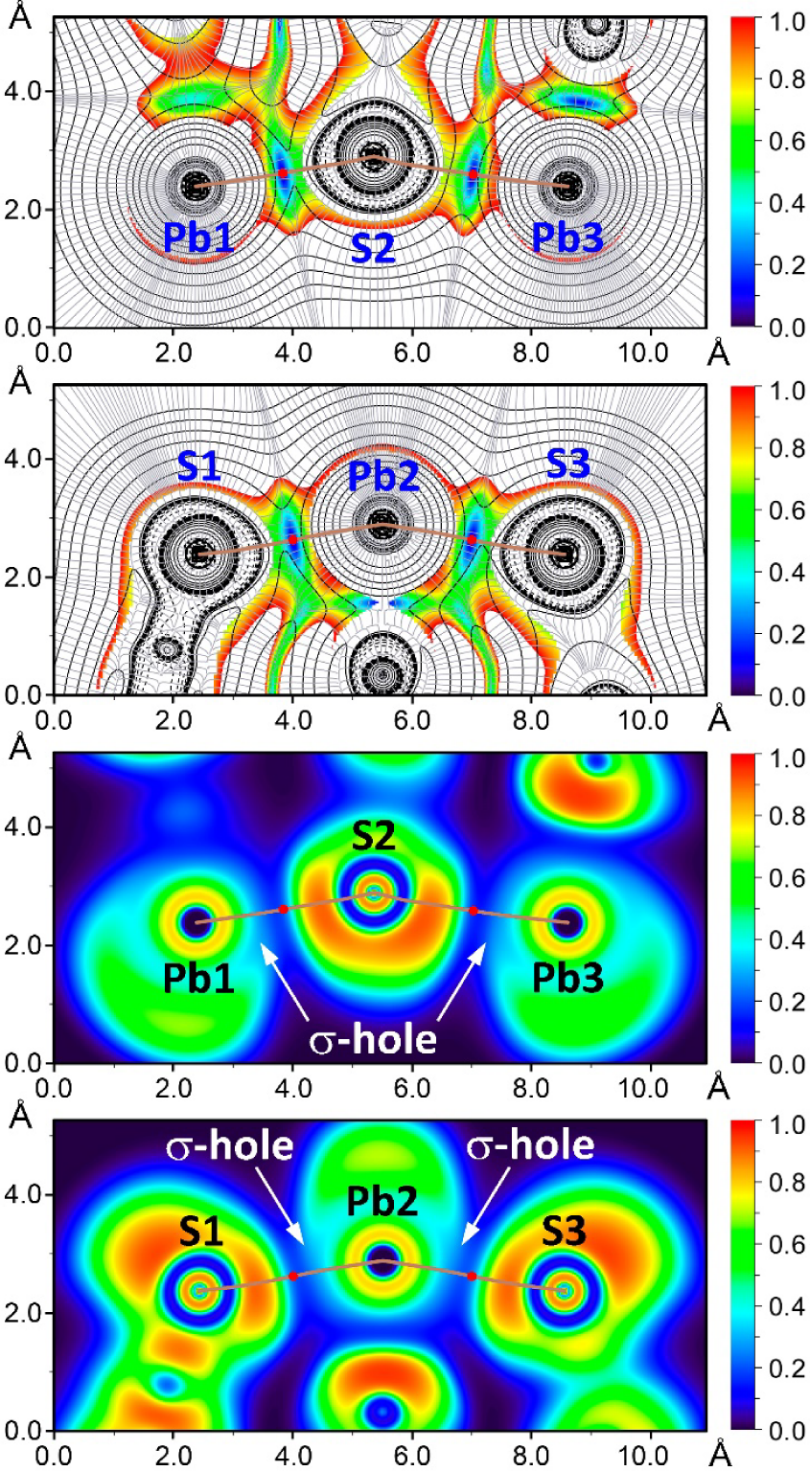
2D plots of the Laplacian (dashed and solid lines for negative and positive values, respectively) including the gradient lines (in gray) overlapped with the 2D RDG maps (two top plots) and 2D ELF maps (two bottom plots) for **1**. The bond paths are represented as brown lines and BCPs of tetrel bonds are shown as red dots. The RDG density cutoff is 0.05 a.u.

The 2D ∇^2^ρ(r) analyses show positive values (represented by solid line isocontours) between the Pb and S atoms (Figure 4). Additionally, 2D RDG maps display blue RDG isocontours in these areas, corresponding to the elongated Pb···S distances typical of noncovalent contacts. The BCPs (bond critical points) that denote tetrel bonds correspond to RDG values near zero. The ELF 2D map provides another level of detail. For instance, in the Pb1···S2···Pb3 triad, it shows a peak in ELF around the S atom (U-shaped red region) and highlights the electrophilic nature of the lead atoms (σ-holes). Furthermore, the bond paths linking the S atom to both Pb1 and Pb3 atoms cross the nucleophilic region of S and the σ-holes at the Pb atoms (Figure 4), thus confirming the σ-hole nature of the TtBs.

The ELF 2D map for the S1···Pb2···S3 triad shows two peaks in ELF around the S1 and S3 atoms, pointing toward the σ-holes at the Pb2 atom. Additionally, the bond paths linking the Pb2 atom to both S1 and S3 atoms cross the nucleophilic regions of S1 and S3 and the σ-holes at the Pb atoms, similar to the other triad. The QTAIM and ELF parameters at the Pb···S BCPs typify the TtBs as intermediate interactions in terms of strength (Table 3). This classification is supported by ρ values between 0.018 and 0.031 a.u., positive and small values of ∇^2^ρ(r), and the greater absolute value of |V(r)| compared to G(r) at these BCPs. Furthermore, negative values of λ_2_ reaching up to −0.024 a.u. indicate the presence of a moderately strong interaction for the Pb2···S1 contact, consistent with the shortest distance of the four Pb···S contacts analyzed in the DFT study.

**Figure 5:**
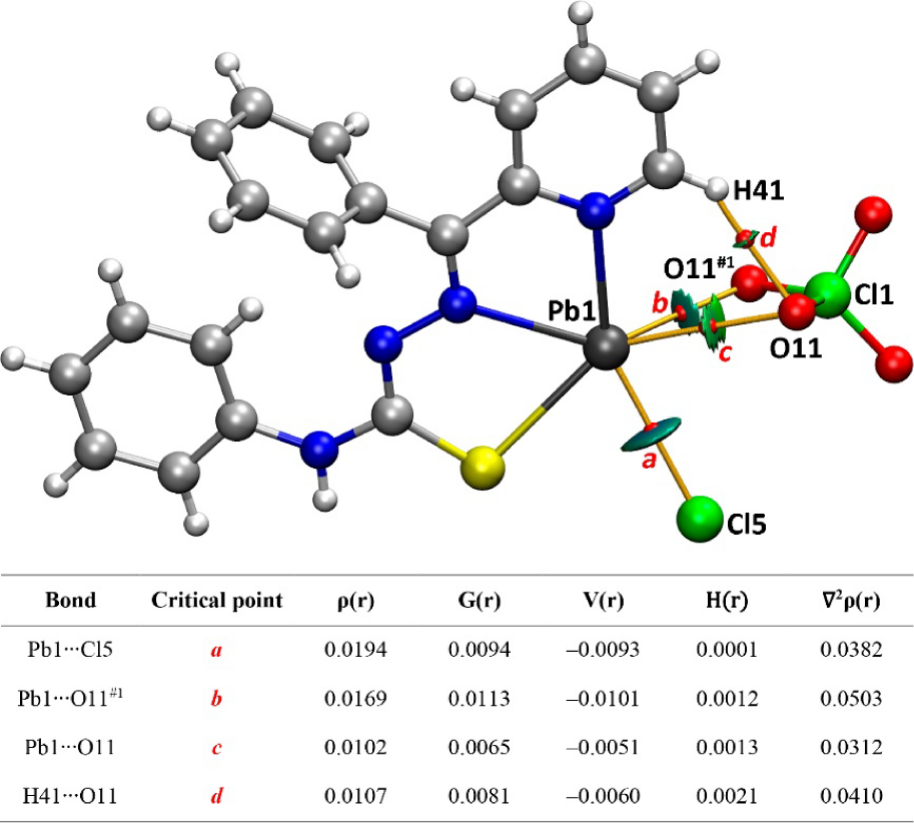
QTAIM (bond critical point = small red spheres, bond paths = orange lines) and NCI plot (RDG = 0.5, ρ cutoff = 0.04, color scale (signλ_2_)ρ = ± 0.03 a.u.) of a trimeric assembly of **1** showing the charge assisted TtBs. Table shows the QTAIM values (a.u.) for the BCPs labeled a–d.

Finally, we have also analyzed the charge-assisted tetrel bonding interactions established between Pb1 and both counterions, viz., chloride and perchlorate (Figures 2, 5 and Table 1). Figure 5 shows the combined QTAIM and NCIplot analysis of the [Pb1(HL)]^+^ cation interacting simultaneously with Cl^–^ and ClO_4_^–^ counterions via charge-assisted tetrel bonding. Specifically, the chloride anion is connected to the Pb atom by the corresponding BCP, bond path, and blue disk-shaped RDG isosurface, characterizing the TtB. The ClO_4_^–^ is linked to the cation by means of three BCPs and bond paths. Two of them connect the oxygen atoms of the perchlorate to the Pb atom, evidencing the formation of a bifurcated charge-assisted TtB. The green color of the RDG isosurfaces characterizing the bifurcated TtBs suggests that the Pb···Cl interaction is stronger. The third BCP connects one O atom of the perchlorate anion to one aromatic hydrogen atom of the ligand, disclosing the formation of an ancillary C–H···O hydrogen bond. The formation energy of this trimer is very large (−123.8 kcal/mol), typical in charge-assisted interactions where Coulombic forces are dominant (ion-pair). Figure 5 also provides information on the QTAIM parameters of the BCPs, showing the typical range of noncovalent interactions with small values of ρ(r) (<0.020 a.u.) and positive values of H(r).

## 4 Conclusions

In conclusion, we report synthesis and characterization of the unprecedented nanosized porous supramolecular nonanuclear complex [Pb_9_(HL)_12_Cl_2_(ClO_4_)](ClO_4_)_3_·15H_2_O·*a*(solvent) (**1**·15H_2_O*·a*(solvent)), which was readily synthesized by electrochemical oxidation of a Pb anode (ambient conditions) in a CH_3_CN:MeOH solution of *N*′-phenyl(pyridin-2-yl)methylene-*N*-phenylthiosemicarbazide (**H**_**2**_**L**), containing [N(CH_3_)_4_]ClO_4_ as a current carrier. The supramolecular cationic cluster **1**^3+^ is constructed from three different species, namely a trinuclear cation [Pb_3_(HL)_3_Cl]^2+^ of a triangle shape, three mononuclear neutral species [Pb(HL)_2_] and three mononuclear cations [Pb(HL)]^+^. In the triangle cationic species each of three space gaps is filled by one [Pb(HL)_2_] and one [Pb(HL)]^+^ species, all together yielding a cyclic supramolecular architecture with a void in the central part. The latter is further filled by an additional Cl^–^ anion, which is located in the middle part of this void, and one ClO_4_^–^ anion, which “seals” this void. In each cationic species [Pb_3_(**HL**)_3_Cl]^2+^ and [Pb(HL)]^+^, as well as in one of the ligands in the neutral species [Pb(HL)_2_], the anionic ligands **HL** covalently bond the Pb^2+^ ions via the pyridine and imine N atoms, as well as the thiocarbonyl S atom. In [Pb(HL)_2_], the second ligand connects to the metal cation via two covalent bonds from the pyridine and imine nitrogen atoms and one tetrel bond from the thiocarbonyl sulfur atom. The supramolecular assembly of **1**^3+^ is stabilized by multiple Pb···S TtBs involving the thiocarbonyl sulfur atoms of neighboring species, Pb···Cl TtBs with the central Cl^–^ anion, and Pb···O TtBs with the three O atoms of the ClO_4_^–^ ion. The noncovalent σ-hole characteristics of the intermolecular Pb···S interactions in the trimeric assembly were confirmed through DFT calculations, utilizing 2D plots of RDG maps, ∇^2^ρ(r) and ELF properties, all of which demonstrated the attractive nature of these interactions.

A comparison of the FTIR and ^1^H NMR spectra of **1**·15H_2_O·*a*(solvent) and its parent ligand **H**_**2**_**L** strongly indicates the presence of the deprotonated ligand **HL** upon coordination with the metal cation. In methanol, the solution of **1**·15H_2_O·*a*(solvent) absorbs up to approximately 500 nm due to intraligand and ligand-to-metal transitions. This solution was emissive, displaying a broad emission band ranging from around 420 to 600 nm. The CIE-1931 chromaticity coordinates of (0.33, 0.24) fall within the white region of the chromaticity diagram, confirming that the complex acts as a single-component white light-emitting phosphor. Further investigation is required to fully understand the origin of this emission. The Kubelka–Munk spectrum shows absorption bands extending up to about 625 nm, with estimated band gaps of 2.38 eV (direct) and 1.90 eV (indirect).

Finally, the molecular structure of **1** differs significantly from that recently reported by us [Pb_2_(HL)_2_(CH_3_CN)(ClO_4_)_2_]·2H_2_O (**2**·2H_2_O), which was obtained using a conventional synthetic procedure by reacting Pb(ClO_4_)_2_ with **H**_**2**_**L** in the same CH_3_CN:MeOH solution, thus highlighting a crucial role of the electrochemical conditions.
